# Diet in Relation to the Teeth

**Published:** 1919-09

**Authors:** 


### DIET IN RELATION TO THE TEETH

The importance which the so-called focal infections have lately assumed as sources of disease has brought new prominence to the relation of the teeth to such infections. Indeed, pyorrhea alveolaris has tended to overshadow the far more widespread defects of carious teeth in which the conspicuous damage penetrates rather than surrounds these structures. If further evidence of the almost universal incidence of dental caries were needed, the records of the systematic examination of pupils in the public schools would testify to the degree of prevalence of teeth defects. The underlying causes are still unknown. They have usually been sought in local bacteriologic conditions within the mouth, if one may conclude from the emphasis that dentists place on oral hygiene. They seem not to realize that the presence of bacteria in itself never causes disease: we have millions of bacteria in our intestines and still thrive. What harm do the micro-organisms do in the month?

Evidently much depends on our powers of resistance. Disease of many sorts is the outcome of the presence of micro-organisms along with the lack of resistance or immunity factors. Facts in harmony with such a hypothesis seem to have been brought to light through the recent researches on scurvy. Zilva and Wells,[1] who have had

[1] Zilva, S. S., and Wells, F. M.: Changes in the Teeth of the Guinea-Pig, Produced by a Scorbutic Diet, proc. Roy. Soc., B 90: 505, 1919.

unusual opportunities for studying scorbutic animals at the Lister Institute in London, have convinced themselves that the mildest degree of scurvy which could just be discovered at the post-mortem examination produced well-defined changes in the structure of the teeth; and in their numerous examinations they have not observed a single exception to this statement. In advanced cases of scurvy, in guinea-pigs and monkeys, the teeth were apparently sound, but useless, since they had been loosened by the gradual absorption of the cement membrane of the alveolar sockets, which had left exposed that portion below the neck. As a result, Zilva and Wells state there must have occurred that periostitic pain or something analogous which follows in the case of human patients who are suffering from shrunken alveoli. These teeth also presented, in addition, all the appearances of the changes of senility.

The British observers regard the most striking changes in the teeth brought about by a scorbutic diet as a sort of fibroid degeneration which they designate as "fibrosis." It is believed that the characteristic structural metamorphoses in the dental pulp are not dependent on an inflammatory condition, but are referable directly to an altered metabolism or to constitutional changes due to diet. In the complete "pulpar fibrosis" of Zilva and Wells, "no trace of cellular organization, no trace of cell nuclei, no trace of interstitial cement substances can be found anywhere. Nerves, cells, blood vessels and odontoblasts have all shared the process of fibrification and are no longer recognizable. The fine cellular connective tissue, which is but a loose mass of network in the normal state, has either become grossly hypertrophied or quite obliterated, and its place taken by a new, firm, fibrous structure, devoid of cells, nuclei, or any regular arrangement of constitutional parts." In a scurvy tooth, the condition persists right up to the apex of the root; the change appears to start first in the odontoblastic cells at the top of the pulp, working down toward the apex, followed by distended blood vessels and hemorrhage; then complete fibroid degeneration follows.

If we may believe the new evidence, which deserves a respectful hearing, the teeth are among the parts of the organism first affected by scorbutic causes. Damage may be detected in the teeth before any other symptoms are discovered. For this reason it is not at all unlikely that tooth defects arise owing to faulty diets that are not clearly recognized beforehand. Zilva and Wells remind us of the contention of Hess[2] of New York, that children not infrequently suffer from a "latent scurvy" which is a state of malnutrition that can be diagnosed only by the improvement in the state after the administration of an antiscorbutic. If such unrecognized conditions are widely prevalent, they may help to elucidate the hitherto unexplained prevalence of tooth decay, and they furnish an added argument for renewed efforts to make human dietaries as wholesome as our scientific knowledge, supplemented by food instincts, will permit.—*Journal A. M. A.*, Aug. 9, 1919.

